# Ammonia in Cocaine Poisoning

**Published:** 1890-02

**Authors:** 


AMMONIA IN COCAINE POISONING.—A case of poisoning by a very moderate quantity of cocaine is reported by Dr. Golovkoff, in the proceedings of the Caucasian Medical Society, where ammonia was used with good effect to restore the patient. The patient was a somewhat delicate woman, who was suffering severely from toothache. The pain becoming unbearable, Dr. Golovkoff injected fifteen minims of a two per cent. solution of hydrochlorate of cocaine under the skin of the left cheek, which gave relief for three or four hours, when the pain returned as acutely as ever. A second fifteen minims were injected, and in about five minutes' time the patient became restless, her pupils dilated, the surface of the skin became pale, the pulse and likewise the respiration became rapid, and shivering came on. A curious effect, too, was produced on the sounds of the heart, causing them to be audible at the distance of two paces from the patient. There was great pain over the cardaic region and back, together with a dread of death and convulsive movements of the limbs. There was some liquor ammoniæ at hand, and this the patient was given to smell, and a few drops were given internally every five or ten minutes. Amyl nirrite was also employed, but the latter seemed to do more harm than good, while the ammonia soon brought the pulse and respiration, and indeed the general condition of the patient, into something more like their natural condition, so that in about a couple of hours she had quite recovered.—*Lancet, November 30th, 1889.*

